# Effects of an aerobic training program on liver functions in male athletes: a randomized controlled trial

**DOI:** 10.1038/s41598-023-36361-4

**Published:** 2023-06-09

**Authors:** Mohd Arshad Bari, Mohammed Aiyad MahmoodAlobaidi, Hena Ayub Ansari, Junaid Ahmad Parrey, Arish Ajhar, Shibili Nuhmani, Ahmad H. Alghadir, Masood Khan

**Affiliations:** 1 Department of Physical Education, Aligarh Muslim University, Aligarh, India; 2 Department of Pathology, JN Medical College, Aligarh Muslim University, Aligarh, Uttar Pradesh India; 3 Department of Physical Therapy, Imam Abdulrahman Bin Faisal University, Dammam, Eastern Province Saudi Arabia; 4 Department of Rehabilitation Sciences, College of Applied Medical Sciences, King Saud University, Riyadh, Saudi Arabia

**Keywords:** Rehabilitation, Liver

## Abstract

The optimal functioning of the liver is essential for athletic performance. It is necessary to maintain the liver’s enzymes at an optimal level so that liver cells can be protected from inflammation or damage. This study investigated the effects of a 12-week aerobic exercise program on the liver function of adult athletes. A pretest–posttest experimental design was used. A total of thirty healthy male athletes (football players) aged 21 to 24 years were recruited for this study and randomly and equally divided into the experimental group (EG) and control group (CG). The CG did not participate in any special activities. The EG performed an aerobic training program consisting of several exercises for 12 weeks. Evaluation of all participants in both groups was carried out before and after the intervention by measuring the blood levels of Alkaline phosphate, AST/SGOT, ALT/SGPT, Bilirubin Total/indirect/direct, Albumin, Globulin, and Total protein using the standard methods by collecting blood samples. There was a significant decrease (*p* < 0.05) in Bilirubin and globulin levels in the EG after 12 weeks of aerobic training sessions. However, there was no significant difference in alkaline phosphate, AST/SGOT, ALT/SGPT Total protein, and Albumin (*p* > 0.05) between both groups post-treatment. The 12 weeks of aerobic training used in the study can potentially improve the liver function of adult athletes.

## Introduction

The liver carries out the detoxification of various metabolites, the production of digestive enzymes and the synthesis of proteins, which is situated in the right upper quadrant of the body and beneath the diaphragm. In addition, the liver’s functions are the metabolism, control of red blood cells (RBCs), and production and storage of glucose. The liver serves as an engine for the body, carrying out several tasks to keep other bodily systems running smoothly. Optimal functioning of the liver is necessary for good athletic performance. To prevent liver cells from inflammation and damage, it is necessary to maintain the liver's enzymes at an optimal level. Liver malfunction can have adverse effects on athletic performance. The disbalance of enzymes of the liver in athletes can lead to different acute or chronic liver diseases^1^. In coordination with other vital organs, the liver performs various functions, giving athletes extra push to achieve their best performance. As the liver is interconnected with other systems and vital organs of the body, it means malfunctioning of the liver can impact other organs as well, resulting in deterioration of athletic performance.

The liver mainly controls the metabolism of lipids and glucose. If other underlying factors, such as alcohol consumption, are ruled out, a liver fat percentage greater than 5.6 percent is considered abnormal, referred to as non-alcoholic fatty liver disease (NAFLD)^2^. NAFLD is widespread; it is the most frequent cause of increased liver enzymes in many developed and developing countries^3^. NAFLD is intimately linked to obesity and type 2 diabetes^4,5^. NAFLD is prevalent in roughly 70% of patients with type 2 diabetes. Because NAFLD may lead to steatohepatitis, cirrhosis, and hepatocellular cancer, proper treatment is critical^5^. Furthermore, NAFLD is linked to cardiovascular disease and mortality^6^.

One of the liver’s primary functions is to break down old or damaged RBCs, which results in the production of bilirubin. This molecule is then mixed with others to form bile, an important digestive fluid^7^. Since only glucose and not fat can be metabolized by some organs, such as erythrocytes and the brain, the liver is crucial in maintaining stable blood glucose levels during a fasting.

Activities that entail repeated, large muscle movements are known as aerobic exercises. These activities boost oxygen inhalation, speed up oxygen transport to the liver and other important organs by altering breathing patterns and increasing the heart rate^8,9^. Any physical activity or exercise improves liver function in several ways^10,11^. Our circulatory system is strengthened by aerobic exercises, especially the heart muscles, which make it easier for the heart to pump blood. This causes a slowdown of the heartbeat and increases blood flow, which makes it easier for the liver to filter blood and return it to the bloodstream. To the best of the authors’ knowledge, no study has evaluated the effects of a 12-week-long aerobic training program on liver function in male athletes. Therefore, this research aimed to investigate the effects of a 12-week-long aerobic training program on the liver function of adult athletes. We hypothesized that this aerobic training program significantly affects the liver functions measured through blood tests in healthy male football athletes. The football players were selected in our study as football is one of the most popular sports worldwide and is associated with high-intensity physical activity and energy expenditure. Moreover, football players may be at an increased risk for liver function abnormalities due to the high levels of oxidative stress and inflammation associated with intense physical activity. For instance, a study by Ekun et al.^12^ and Rengers et al.^13^ found that football players had significantly higher levels of liver enzymes.

## Methods and material

### Participants

**Figure 1:**
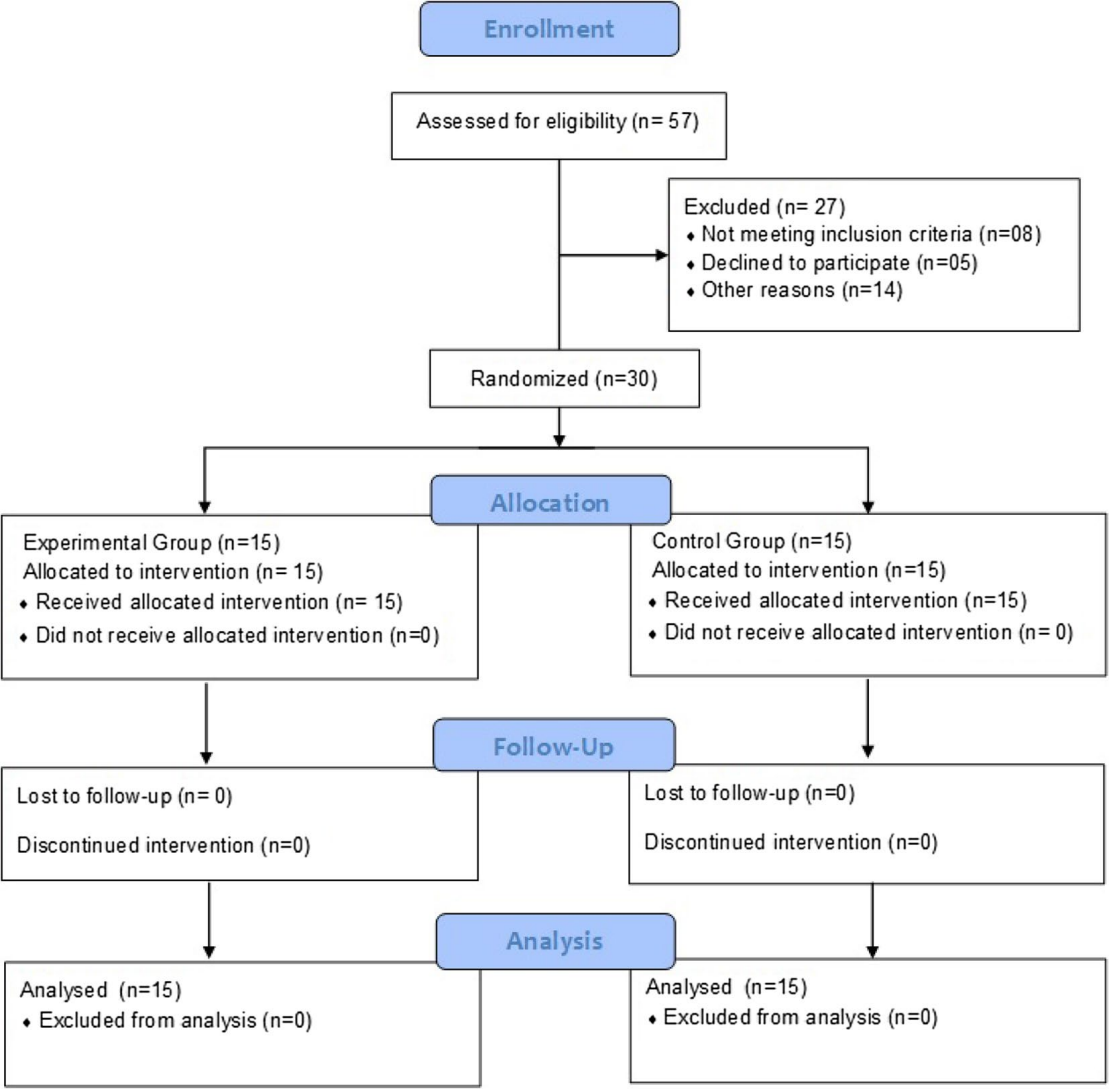
Consolidated standards of reporting trials (CONSORT) flowchart showing the number of participants assessed for eligibility, randomized and analyzed during the study.

This study used a pretest–posttest experimental design. The sample size was estimated as 14 in each group by using the G* power software V 3.1.9.2 based on a previous study which compared the efficacy of aerobic training and resistance training on liver enzyme levels in patients with non-alcoholic fatty liver using the variable ALT, effect size 0.98, an alpha level of 0.05 and power (1- beta) of 0.8^14^. One participant was added to each group to avoid attrition bias which made a total of 30 participants for the study. Therefore, thirty healthy male athletes (football players) aged 21 to 24 years were chosen from different stadiums in New Delhi (India) for this purpose. Liver functions may differ between younger and older individuals due to changes in metabolism and exposure to environmental factors over time. Therefore, by focusing on a specific age group (21 to 24 years) of football players, this study aimed to control for potential confounding variables related to age and to provide a more focused investigation into the effects of aerobic training on liver function in this population. Participants performed interventions in the same stadiums. Participants were recruited from September 2021 to July 2022. Figure 1 shows the number of assessed, recruited, randomized, and analyzed participants. These were equally and randomly divided into two groups, with 15 participants in each group. Participants with a history of severe illness, surgery, or damage to the liver, fatty liver disease, cirrhosis, blood infections, neuromuscular conditions, or the presence of any disorders such as fever, high blood pressure, high blood sugar, etc., were excluded from the study. Randomization was performed by an independent researcher who was not associated with the study, using the software SPSS, version 20 (SPSS Inc., Chicago, IL, USA), and the lottery method.

The outcome assessor was also blind to the randomization/allocation of participants. The first group, the aerobic group, was designated the "experimental group (EG)," whereas the second group was named the "control group (CG)." Before inclusion into the study, the risk and benefits of the study were discussed with every participant and written informed consent was taken from them. The study was presented to the institutional review board of Aligarh Muslim University, Aligarh, India, which approved it (ID: DN 4900/FSS on 1/9/2021). The study complied with “The Code of Ethics of the World Medical Association (Declaration of Helsinki)”. All methods were performed in accordance with the relevant guidelines and regulations. The study has been registered retrospectively in the protocol registration and results system (clinicaltrials.gov, ID: NCT05704608 on 30/01/2023).

### Experimentation

The CG was under rigorous supervision and did not participate in any structured exercise program besides their regular soccer practice. The EG was well-versed in their assigned training program and performed the experimental procedure only for 12 weeks. Both groups were allowed to practice their regular soccer practice. Evaluation of all patients in both EG and CG was carried out before and after the treatment program by measuring the levels of (Alkaline phosphate, AST/SGOT, ALT/SGPT, Bilirubin Total/indirect/direct, Albumin, Globulin and Total protein) respectively^15,16^ using standard methods (Autoanalyzer, Mindray BS 800, China) by collecting Blood samples, 5 mls each of baseline into lithium heparin containers for estimation of above biochemical parameters at the NABL accredited pathology laboratory.

### Training program

The aerobic exercise program which was given to the EG was a series of treadmill exercises, including slow, medium, and fast walking; walking with a 30° incline (uphill); walking with a 30° declination (downhill); jogging with a 30° incline (uphill); running with a 30° declination (downhill); and cycling^17–19^. The week-wise details of the type of exercises, duration, number of sets, and rest periods are given in Supplementary Tables 1, 2 and 3. The training session lasted for around 50 min and was performed 5 days a week. The training frequency and intensity were never maximized. The identical load, which varied in volume, intensity, and frequency, was applied to every participant. By periodically tracking heart rate, the researcher could keep an eye on how intense the training intensity was using this technique.

### Outcome measures

Blood levels of the following substances were taken as outcome measures.

- Bilirubin-total mg/dL,
- Serum AST/SGOT (U/L),
- Serum ALT/SGPT (U/L),
- Alkaline phosphatase (U/L),
- Protein-total (g/L),
- Albumin (g/dL),
- Globulin (Gm/dL).

### Analysis of data

The data of 30 participants (15 in each group) were analyzed using the SPSS statistical software version 20 (SPSS Inc., Chicago, IL, USA). Liver function was analyzed for both pre-test and post-test of all the participants. For 12 weeks, the EG did their assigned activities, which were designed as an aerobic training program. After 12 weeks, post-tests on the dependent variables mentioned above were administered to the participants, resulting in final scores. The difference between the initial and final scores determined the impact of the aerobic training program on selected variables. A paired sample t-test and Cohen's d were used to investigate statistical significance with impact size. To test the hypothesis of this study, 0.05 levels were used.

## Results

### Description of the subjects

Table 1 displays descriptive statistics of the age, height, and weight of adult athletes who were recorded before and after a specifically designed aerobic training program. The mean value of EG’s age was 22.47 years (SD 1.13), the height was 173.33 cm (SD 4.79), and the weight was 74.47 ± 05.83 kg (Pre) and 73.87 ± 5.54 kg (Post), and CG’s age was 22.20 years (SD 1.15), the height was 170.67 cm (SD 3.66), and the weight was 73.27 ± 8.03 kg (Pre) and 73.47 ± 8.29 kg (post) respectively.

**Table 1:** Description of the participants.

Groups	N	Age (years)		Height (cm)		Weight (Kg)	
		Mean	SD	Mean	SD	Mean	SD
EG	15	22.47	1.13	173.33	4.79	74.47	5.83
CG	15	22.20	1.15	170.67	3.66	73.27	8.03

*EG* experimental group, *CG* control group, *SD* standard deviation.

Table 2 shows descriptive data for adult athletes’ bilirubin-total mg/dL, serum AST/SGOT (U/L), serum ALT/SGPT (U/L), alkaline phosphatase (U/L), protein total (g/L), albumin (g/dL), and globulin (Gm/dL) before and after participating in a specifically designed aerobic training program. Bilirubin-Total mg/dL in EG was 1.38 ± 0.14 (pre) and 1.28 ± 0.13 (post), whereas Bilirubin-Total mg/dL in CG was 1.31 ± 0.06 (pre) and 1.22 ± 0.30 (post). Serum AST/SGOT (U/L**)** in EG was 36.53 ± 10.68 (pre) and 38.00 ± 9.11 (post), whereas Serum AST/SGOT (U/L) in CG was 41.27 ± 3.65 (pre) and 41.40 ± 4.23 (post). Serum ALT/SGPT (U/L) in EG was 31.53 ± 6.08 (pre) and 32.73 ± 5.62 (post), whereas serum AST/SGOT (U/L) in CG was 35.73 ± 3.61 (pre) and 34.87 ± 3.33(post). Alkaline Phosphatase (U/L) in EG was 79.00 ± 13.88 (pre) and 82.73 ± 12.33 (post), whereas Alkaline Phosphatase (U/L) in CG was 78.40 ± 5.71 (pre) and 78.53 ± 3.73 (post). Protein total (g/L) in EG was 7.81 ± 0.80 (pre) and 7.63 ± 0.76 (post), whereas protein total (g/L) in CG was 7.77 ± 0.43 (pre) and 7.57 ± 0.37 (post). Albumin (g/dL) in EG was 4.46 ± 0.27 (pre) and 4.31 ± 0.26 (post), whereas albumin (g/dL) in CG was 4.55 ± 0.47 (pre) and 4.52 ± 0.22 (post). Globulin (Gm/dL) was 2.590.19 (pre) and 2.500.16 (post) in EG, and 2.57 0.09 (pre) and 2.560.10 (post) in CG.

**Table 2:** Descriptive data for adult athletes’ Bilirubin-total mg/dL, Serum AST/SGOT (U/L), Serum ALT/SGPT (U/L), Alkaline phosphatase (U/L), Protein total (g/L), Albumin (g/dL), Globulin (Gm/dL).

	Groups		Mean	Std. deviation	Std. error mean
Bilirubin-total mg/dL	EG	Pre	1.38	0.14	0.03
		Post	1.28	0.13	0.03
	CG	Pre	1.31	0.06	0.01
		Post	1.22	0.30	0.08
Serum AST/SGOT (U/L)	EG	Pre	36.53	10.68	2.75
		Post	38.00	9.11	2.35
	CG	Pre	41.27	3.65	0.94
		Post	41.40	4.23	1.09
Serum ALT/SGPT (U/L)	EG	Pre	31.53	6.08	1.57
		Post	32.73	5.65	1.45
	CG	Pre	35.73	3.61	0.93
		Post	34.87	3.33	0.86
Alkaline phosphatase (U/L)	EG	Pre	79.00	13.88	3.58
		Post	82.73	12.33	3.18
	CG	Pre	78.40	5.71	1.47
		Post	78.53	3.73	0.96
Protein total (g/L)	EG	Pre	7.81	0.80	0.20
		Post	7.63	0.76	0.19
	CG	Pre	7.77	0.43	0.11
		Post	7.57	0.37	0.09
Albumin (g/dL)	EG	Pre	4.46	0.27	0.07
		Post	4.31	0.26	0.06
	CG	Pre	4.55	0.47	0.12
		Post	4.52	0.22	0.05
Globulin (Gm/dL)	EG	Pre	2.59	0.19	0.05
		Post	2.50	0.16	0.04
	CG	Pre	2.57	0.09	0.02
		Post	2.56	0.10	0.02

*EG* experimental group, *CG* control group.

The mean differences in bilirubin-total mg/dL, serum AST/SGOT (U/L), serum ALT/SGPT (U/L), alkaline phosphatase (U/L), protein total (g/L), albumin (g/dL), and globulin (Gm/dL) between adult athletes' pre- and post-aerobic training programs were compared using a paired sample *t* test. Table 3 results show that variables like bilirubin-total mg/dL (t = 3.41, p = 0.00, p 0.05) and globulin (Gm/dl) (t = 2.29, p = 0.03; p 0.05) of the EG have statistically significant mean differences between the pre-test and post-test of an aerobic training program. Whereas for the CG, t = 1.22, p = 0.24, p > 0.05) and (t = − 0.73, p = 0.47, p > 0.05, show statistically insignificant mean differences between the pre-test and post-test of an aerobic training program. The value of the bilirubin-total mg/dL of the EG, Cohen’s *d,* was 0.74 > 0.50, which indicated a medium effect size, and the CG, Cohen’s *d,* was 0.41 < 0.50, which indicated a small effect size. The value of Cohen's d for the EG was 0.51 > 0.50, which means that the group had a medium effect size. For the CG, the value was 0.10 < 0.20, meaning they had no effect size. The result of the table reveals that variables such as serum AST/SGOT (U/L), serum ALT/SGPT (U/L), alkaline phosphatase (U/L), protein total (g/L), and albumin (g/dL) of both EG and CG show statistically insignificant mean differences between the pre-test and post-test of an aerobic training program.

**Table 3:** Comparison of Bilirubin total mg/dl, AST/SGOT u/l, ALT/SGPT u/l, Alkaline phosphate u/l Protein total g/l, Albumin g/dl, Globulin (Gm/dL) mean between pre and post-aerobic training program.

Variables		Groups	Paired differences			t	p-value	Cohen’s d
			Mean difference	SD	Std. error mean			
Bilirubin-total mg/dL	Pre test–post test	EG	0.09	0.10	0.02	3.41	0.00*	0.74
	Pre test–post test	CG	0.09	0.29	0.07	1.22	0.24	0.41
Serum AST/SGOT (U/L)	Pre test–post test	EG	− 1.46	12.01	3.10	− 0.47	0.64	0.14
	Pre test–post test	CG	− 0.13	4.15	1.07	− 0.12	0.90	0.03
Serum ALT/SGPT (U/L)	Pre test–post test	EG	− 1.20	7.28	1.88	− 0.63	0.53	0.20
	Pre test–post test	CG	0.86	3.87	0.99	0.86	0.40	0.24
Alkaline phosphatase (U/L)	Pre test–post test	EG	− 3.73	18.729	4.83	− 0.77	0.45	0.28
	Pre test–post test	CG	− 0.13	06.034	1.55	− 0.08	0.93	0.02
Protein total (g/L)	Pre test–post test	EG	0.182	0.68	0.17	1.02	0.32	0.23
	Pre test–post test	CG	0.207	0.42	0.11	1.88	0.08	0.49
Albumin (g/dL)	Pre test–post test	EG	0.14	0.35	0.09	1.60	0.13	0.56
	Pre test–post test	CG	0.03	0.34	0.08	0.38	0.70	0.08
Globulin (Gm/dL)	Pre test–post test	EG	0.09	0.15	0.04	2.29	0.03*	0.51
	Pre test–post test	CG	0.01	0.09	0.02	− 0.73	0.47	0.10

*EG* experimental group, *CG* control group.

df = 14.

*p < 0.05.

## Discussion

The training effect is the physiological changes that occur due to frequent involvement in exercise programs. The nature of training programs has its effects, which means different training programs produce different kinds of effects on physiological and biochemical parameters. The optimal training programs are one that quickly increases the desired quality while minimizing undesirable repercussions. We've been curious for a long time about how much exercise and what forms (modes) are optimal for achieving health benefits^20^. No one quantity or exercise style is likely ideal for every health benefit. This study examined how an aerobic exercise training program affected the liver function of adult athletes. Our key findings imply that aerobic exercise training enhances several of the previously mentioned outcome indicators. Table 1 indicated that the weight (kg) of the EG was reduced by 0.80%, but the weight of the CG grew by 0.27%, with no impact size. The findings showed that aerobic exercise caused a significant increase in muscle mass.

The EG’s total bilirubin mg/dL was reduced by 7.24 percent (1.38 mg/dL to 1.28, normal range of 0.2–1.30) with a medium effect size, whereas that of the CG decreased by 6.8 percent with no impact size. The study's results indicated that following the training program, total bilirubin levels were restored to their normal range. A low-intensity aerobic training program proved ineffective in raising total bilirubin level because total bilirubin is mainly determined by the degree of hemolysis, which suggests that the intensity of exercise primarily determines total bilirubin.

The reductions in total bilirubin concentrations seem to be linked to changes caused by 12 weeks of aerobic exercise. The upper limit of the reference range for total bilirubin levels in the athletes was determined to be 0.2–1.3 mg/dL. Following exercise, bilirubin levels increased, similar to Swift et al.^18^ study on the influence of different doses of aerobic exercise training on total bilirubin levels.

After 12 weeks of aerobic activity, the liver enzymes aspartate serum AST/SGOT (U/L), ALT/SGPT (U/L), and alkaline phosphatase (U/L) slightly increased. The researchers noted that the EG’s protein total (g/L) decreased insignificantly by 2.30 percent with a small effect size, whereas the CG’s decreased by 2.57 percent with a small effect size. Albumin (g/dL) insignificantly reduced by 3.36 percent in the EG, with a medium impact size, but significantly by 0.65 percent in the CG, with a small effect size. The EG’s globulin (Gm/dL) fell 3.47 percent with a medium effect size, whereas the CG’s dropped 0.38 percent with no impact size.

After 12 weeks of aerobic activity, the liver enzymes aspartate serum AST/SGOT (U/L), ALT/SGPT (U/L), and alkaline phosphatase (U/L) slightly increased. Serum AST/SGOT (U/L) increased insignificantly by 4.02 percent with no impact size in the EG, whereas it increased by 0.31 percent with no effect size in the CG. The EG’s serum ALT/SGPT (U/L) improved insignificantly by 3.80% with a low impact size, whereas the CG’s dropped by 2.40% with a small effect size. Alkaline phosphatase (U/L) got elevated in the EG by 7.72 percent with a small effect size, whereas in the CG, it went up by 0.16 percent with a small impact size.

Studies by Gutierrez-Grobe et al.^21^, Johnson et al.^22^, and Farzanegi et al.^23–25^ indicated a reduction in liver enzyme levels^21–23^. Farzanegi et al.^23^ reported that 6 weeks of aerobic exercise decreased ALT, AST, and ALP enzymes but had no effect on lipid levels, which contradicts our results^23^. Aerobic exercise at 50–70 percent of one's maximum heart rate for 8 weeks can treat fattening liver disease in men, and this might also aid in lowering ALT and AST blood levels^26^. Contradictory to the results of the present study, El-Kader et al.^27^ showed that aerobic exercise decreased ALT, ALP, AST, and GGT concentrations. The study found that AST, ALT, and alkaline phosphatase levels did not significantly change between pre-and post-training programs. These slightly elevated but still within the normal range AST, ALT, and alkaline phosphatase readings compared to pre-examination appear to be caused by changes made by 12 weeks of aerobic activity. This study’s 12-week aerobic exercise program resulted in negligible changes in liver enzymes that might have been caused by a slight decrease in enzyme use or changes in body weight.

According to the study, the EG’s protein total (g/L) decreased by an inconsequential 2.30 percent with a negligible impact size. The drop in the CG was 2.57 percent; however, it had a modest impact size. In the EG, albumin (g/dL) decreased insignificantly by 3.36 percent with a medium impact size, whereas it declined by 0.65 percent with a small effect size in the CG. Globulin (Gm/dL) decreased by 3.47 percent with a moderate effect size in the EG, whereas it decreased by 0.38 percent with no impact size in the CG.

The difference between the mean albumin levels of young athletes before and after exercise was not statistically significant (p > 0.05). Both numbers are falling below the average. This suggests that the study's subject pool consisted of healthy individuals. The current study’s findings do not agree with other studies, which demonstrated that exercise considerably raises plasma albumin levels. After exercise, the quantity of plasma albumin in the blood rises during the first hour of recovery. As a result, the most probable mechanism for this response is albumin redistribution from the interstitial to the intravascular region^28,29^. Increased lymph flow during and after exercise may lead to albumin redistribution. In addition, within a few hours following exercise, the primary contributors to increased lymph flow and muscle pumping should return to normal. In Nagashima et al. study^29^, it was anticipated that the spike in blood albumin following exercise would revert to normal in 24 h if there were no other means to compensate for it.

Our study has some limitations. First, our investigation was a study containing just one EG; consequently, we cannot confirm that the outcomes of the study were totally attributed to the selected, designed exercise program as there were no instructions given on a diet, which has a huge role to play in the functioning of the liver. Therefore, more research involving another EG is necessary to verify the interaction effects. Second, there was a small sample size of healthy participants, and the external validity of the current results has to be increased by more research with larger sample sizes. Third, the participants in our study were advised to continue their normal regular diet during the time of the study. They were advised not to consume any additional dietary supplements at the time of the study. However, we didn’t monitor the eating habits and diet of the participants. Eating habits and diet may have an influence on the liver enzyme level. Finally, because the study subjects were healthy adults, it may be difficult to generalize our findings to other problems. Thus, further study will be focused on differences in type, duration, and intensity of aerobic exercise on the liver and its functions in athletes.

## Conclusion

In conclusion, the current study shows that a selected aerobic training program has a good impact on some biochemical variables of the liver, such as bilirubin and globulin, in a healthy adult male athlete. The 12 weeks of aerobic training used in the study can potentially improve the liver function of adult athletes.

## Supplementary Information

Supplementary Tables.

## Data Availability

The data associated with the paper are not publicly available but are available from the corresponding author on reasonable request.

## References

[1] S. Wolfe, Optimal Lab Ranges for Performance Athletes Part 2: Oxygen deliverability, Liver Function, and Kidney Function.

[2] Szczepaniak LS, Nurenberg P, Leonard D, Browning JD, Reingold JS, Grundy S, Hobbs HH, Dobbins RL (2005). Magnetic resonance spectroscopy to measure hepatic triglyceride content: Prevalence of hepatic steatosis in the general population. Am. J. Physiol.-Endocrinol. Metab..

[3] Clark JM, Brancati FL, Diehl AM (2003). The prevalence and etiology of elevated aminotransferase levels in the United States. Am. J. Gastroenterol..

[4] Chalasani N, Younossi Z, Lavine JE, Diehl AM, Brunt EM, Cusi K, Charlton M, Sanyal AJ (2012). The diagnosis and management of non-alcoholic fatty liver disease: Practice Guideline by the American Association for the Study of Liver Diseases American College of Gastroenterology, and the American Gastroenterological Association. Hepatology.

[5] Byrne CD (2012). Dorothy Hodgkin Lecture 2012* Non-alcoholic fatty liver disease, insulin resistance and ectopic fat: A new problem in diabetes management. Diabet. Med..

[6] Ekstedt M, Hagström H, Nasr P, Fredrikson M, Stål P, Kechagias S, Hultcrantz R (2015). Fibrosis stage is the strongest predictor for disease-specific mortality in NAFLD after up to 33 years of follow-up. Hepatology.

[7] C. Clinic, Bilirubin Test, 2023. https://my.clevelandclinic.org/health/diagnostics/17845-bilirubin (Accessed 20 Decenber 2022) (2023).

[8] Garzon RC (2018). Why and How to Do Aerobic Training, Including High-intensity Interval Training.

[9] McDonald DG, Hodgdon JA (2012). The Psychological Effects of Aerobic Fitness Training: Research and Theory.

[10] Smart N, King N, McFarlane J, Graham P, Dieberg G (2018). Effect of exercise training on liver function in adults who are overweight or exhibit fatty liver disease: A systematic review and meta-analysis. Br. J. Sports Med..

[11] Marcinko K, Sikkema SR, Samaan MC, Kemp BE, Fullerton MD, Steinberg GR (2015). High intensity interval training improves liver and adipose tissue insulin sensitivity. Mol. Metab..

[12] Ekun OA, Emiabata AF, Abiodun OC, Ogidi NO, Adefolaju FO, Ekun OO (2017). Effects of football sporting activity on renal and liver functions among young undergraduate students of a Nigerian tertiary institution. BMJ Open Sport Exerc. Med..

[13] Rengers TA, Orr SC, Marks CR, Hew-Butler T, Choi MD, Butcher SJ, Drignei D, Brown EC (2021). Effects of high-intensity interval training protocols on liver enzymes and wellness in women. J. Sports Med..

[14] Shamsoddini A, Sobhani V, Chehreh MEG, Alavian SM, Zaree A (2015). Effect of aerobic and resistance exercise training on liver enzymes and hepatic fat in Iranian men with nonalcoholic fatty liver disease. Hepat. mon..

[15] Thapa B, Walia A (2007). Liver function tests and their interpretation. Indian J. Pediatr..

[16] Giannini EG, Testa R, Savarino V (2005). Liver enzyme alteration: A guide for clinicians. CMAJ.

[17] Bangsbo J, Mohr M, Poulsen A, Perez-Gomez J, Krustrup P (2006). Training and testing the elite athlete. J. Exerc. Sci. Fit.

[18] Swift DL, Johannsen NM, Earnest CP, Blair SN, Church TS (2012). The effect of different doses of aerobic exercise training on total bilirubin levels. Med. Sci. Sports Exerc..

[19] Matos N, Winsley RJ (2007). Trainability of young athletes and overtraining. J. Sports Sci. Med..

[20] Slentz CA, Houmard JA, Kraus WE (2007). Modest exercise prevents the progressive disease associated with physical inactivity. Exerc. Sport Sci. Rev..

[21] Gutierrez-Grobe Y, Ponciano-Rodríguez G, Ramos MH, Uribe M, Méndez-Sánchez N (2010). Prevalence of non alcoholic fatty liver disease in premenopausal, posmenopausal and polycystic ovary syndrome women. The role of estrogens. Ann. Hepatol..

[22] Johnson NA, Sachinwalla T, Walton DW, Smith K, Armstrong A, Thompson MW, George J (2009). Aerobic exercise training reduces hepatic and visceral lipids in obese individuals without weight loss. Hepatology.

[23] Farzanegi P, Pour Amin Z, Habibian M (2014). Changes of liver trans-aminases after a period of selected aerobic training in postmenopausal women. Med. Lab. J..

[24] Hong F, Liu Y, Lebaka VR, Mohammed A, Ye W, Chen B, Korivi M (2022). Effect of exercise training on serum transaminases in patients with nonalcoholic fatty liver disease: A systematic review and meta-analysis. Front. Physiol..

[25] Mohammad Rahimi GR, Attarzadeh Hosseini SR (2022). Effect of aerobic exercise alone or in conjunction with diet on liver function, insulin resistance and lipids in non-alcoholic fatty liver disease. Biol. Res. Nurs..

[26] Davoodi M (2012). The effect of eight weeks selected aerobic exercise on liver parenchyma and liver enzymes (AST, ALT) of fat liver patients. J. Shahrekord Univ. Med. Sci..

[27] El-Kader S, Al-Jiffri OH, Al-Shreef FM (2014). Liver enzymes and psychological well-being response to aerobic exercise training in patients with chronic hepatitis C. Afr. Health Sci..

[28] Gillen CM (1994). Blood Volume Regulation After Exercise and Hemorrhage: The Role of Plasma Albumin Content.

[29] Nagashima K, Mack GW, Haskell A, Nishiyasu T, Nadel ER (1999). Mechanism for the posture-specific plasma volume increase after a single intense exercise protocol. J. Appl. Physiol..

