# Supplementary material for: Effects of an aerobic training program on liver functions in male athletes: a randomized controlled trial
Source: Sci Rep. 2023 Jun 9;13:9427. doi: 10.1038/s41598-023-36361-4 (PMC10256744; doi:10.1038/s41598-023-36361-4)
Supplement: Supplementary file 1 — Supplementary Tables. [file 41598_2023_36361_MOESM1_ESM.docx]

**Supplementary Table 1.** Training schedule for the experimental group (Physical Exercises) for I-V weeks.

| **S.No.** | **Exercises** | **Duration in Minutes** | **Set** | **Rest in sec.** |
| --- | --- | --- | --- | --- |
| 01. | Warm up | 05 | 01 | 30 sec |
| 02. | Brisk Walking | 10 | 01 | 30 sec |
| 03. | Low-pace walking on Treadmill | 05 | 01 | 30 sec |
| 04. | Medium pace walking: on Treadmill | 03 | 01 | 30 sec |
| 05. | Fast pace walking on Treadmill | 03 | 01 | 30 sec |
| 06. | 20^o^ Inclination (uphill) Walk on Treadmill | 03 | 01 | 30 sec |
| 07. | 20^o^ declination (downhill )Walk on Treadmill | 03 | 01 | 30 sec |
| 08. | Low pace running on Treadmill | 03 | 01 | 30 sec |
| 09. | Static Cycle exercises | 05 | 01 | 30 sec |
| 10. | Cool Down | 05 | 01 | 30 sec |

**Supplementary Table 2.** Training schedule for the experimental group (Physical Exercises) for V-X weeks (Exercises on treadmill without external weight).

| **S.No.** | **Exercises** | **Duration in Minutes** | **Set** | **Rest in Minutes** |
| --- | --- | --- | --- | --- |
| 01. | Warm up | 05 | 01 | 30 sec |
| 02. | Brisk Walking | 10 | 01 | 30 sec |
| 03. | Low pace walking on Treadmill | 06 | 01 | 30 sec |
| 04. | Medium pace walking: on Treadmill | 04 | 01 | 30 sec |
| 05. | Fast pace walking on Treadmill | 04 | 01 | 30 sec |
| 06. | 25^o^ Inclination (uphill) Walk on Treadmill | 04 | 01 | 30 sec |
| 07. | 25^o^ declination (downhill )Walk on Treadmill | 04 | 01 | 30 sec |
| 08. | Low pace running on Treadmill | 03 | 01 | 30 sec |
| 09. | Static Cycle Exercises | 05 | 01 | 30 sec |
| 10. | Cool Down | 05 | 01 | 30 sec |

**Supplementary Table 3.** Training schedule for experimental group (Physical Exercises) for X-XII weeks (Exercises on treadmill without external weight).

| **S.No.** | **Exercises** | **Duration in Minutes** | **Set** | **Rest in Minutes** |
| --- | --- | --- | --- | --- |
| 01. | Warm up | 05 | 01 | 30 sec |
| 02. | Brisk Walking | 08 | 01 | 30 sec |
| 03. | Low pace walking on Treadmill | 06 | 01 | 30 sec |
| 04. | Medium pace walking: on Treadmill | 04 | 01 | 30 sec |
| 05. | Fast pace walking on Treadmill | 04 | 01 | 30 sec |
| 06. | 30^o^ Inclination (uphill) Walk on Treadmill | 04 | 01 | 30 sec |
| 07. | 30^o^ declination (downhill )Walk on Treadmill | 04 | 01 | 30 sec |
| 08. | Low pace running on Treadmill | 05 | 01 | 30 sec |
| 09. | Static Cycle Exercises | 05 | 01 | 30 sec |
| 10. | Cool Down | 05 | 01 | 30 sec |
